# The effect of Time-Acupoints-Space Acupuncture on fatigue in postoperative chemotherapy patients with breast cancer: a randomized controlled trial

**DOI:** 10.3389/fonc.2025.1518278

**Published:** 2025-03-03

**Authors:** Xin Chen, Zheng Zuo, Li Li, Liangxian Liu, Xiongying Bao, Ran Song, Yinghao Wang, Lingling Wang, Miansheng Zhu, Yan Wang

**Affiliations:** ^1^ First Clinical Medical College, Yunnan University of Chinese Medicine, Kunming, China; ^2^ Medical College, Qinghai University, Xining, China; ^3^ ARIATAS, Association Pour la Recherche et I’Information de I’Acupuncture Time-Acupoints-Space, College of Acupuncture, Paris, France; ^4^ Second Department of Acupuncture and Moxibustion, Dali Bai Autonomous Prefecture Chinese Medicine Hospital, Dali, China

**Keywords:** time-acupoints-space acupuncture (ATAS), acupuncture, breast cancer, fatigue, PiPer fatigue scale (PFS), inflammatory factors

## Abstract

**Background:**

Breast cancer (BC) is a common malignant tumor in women, and cancer-related fatigue (CRF) is prevalent among breast cancer patients. Time-Acupoints-Space Acupuncture (ATAS) is an acupuncture method different from traditional acupuncture. It combines time acupoints with space acupoints, proposing a new treatment approach. This randomized controlled trial aims to evaluate whether ATAS can improve fatigue in postoperative chemotherapy patients with breast cancer.

**Objective:**

This randomized controlled trial focuses on survivors of postoperative chemotherapy for breast cancer, primarily assessing whether ATAS can reduce fatigue in these patients. Additionally, it reports on the effects of ATAS on sleep, anxiety, depression, and inflammatory factors.

**Methods:**

The researchers randomly assigned 90 postoperative breast cancer patients to the ATAS group (n=30), the sham acupuncture group (n=30), and the waitlist control group (n=30). The primary outcome was the Piper Fatigue Scale (PFS), and the secondary outcomes were the Insomnia Severity Index (ISI), Hospital Anxiety and Depression Scale (HADS), Interleukin-2 (IL-2), Interleukin-6 (IL-6), CD3^+^T, and CD4^+^T. Data analysis was performed using the statistical software SPSS, utilizing descriptive statistics and analytic statistics. The significance level was set at less than 0.05.

**Results:**

The baseline differences in PFS scores among the three groups were not statistically significant (*P* > 0.05). ATAS treatment is superior to sham acupuncture and the waitlist control in improving fatigue (mean difference 4.98, 95% CI 3.96 to 6.00, *P*<0.05). Additionally, secondary outcome analysis shows that the ATAS group has positive effects on ISI, HADS, and inflammatory factors. After the treatment ended, ISI (mean difference 15.17, 95% CI 12.28 to 18.06, *P*<0.05), HADS-A (mean difference 8.63, 95% CI 5.18 to 12.08, *P*<0.05), HADS-D (mean difference 7.80, 95% CI 4.73 to 10.87, *P*<0.05). IL-2(mean difference 20.18, 95% CI 11.51 to 28.85, *P*<0.05), IL-6(mean difference 24.56, 95% CI 7.57 to 41.55, *P*<0.05), CD3^+^T(mean difference 79.03, 95% CI 68.56 to 89.50, *P*<0.05), CD4^+^T(mean difference 42.89, 95% CI 35.14 to 50.64, *P*<0.05).

**Conclusions:**

Our preliminary findings indicate that ATAS effectively improves fatigue in postoperative chemotherapy patients with breast cancer. It also has positive effects on sleep, anxiety, depression, and inflammatory factors. These results suggest that ATAS intervention may be an effective method for alleviating fatigue in breast cancer patients.

**Clinical Trial Registration:**

https://www.chictr.org.cn/showproj.html?proj=21999, identifier ChiCTR17013652.

## Introduction

Breast cancer (BC) is a common malignant tumor worldwide and a leading cause of cancer-related deaths among women, posing a serious threat to women’s health. According to a 2020 epidemiological survey, breast cancer has become the most prevalent cancer worldwide, ranking first in incidence among cancers in 185 countries. The number of new cases reached 2.3 million, accounting for 11.7% of the total cancer incidence, with a mortality rate of 6.9%, resulting in more than 685,000 deaths. It is worth noting that in developing countries, the mortality rate of breast cancer is higher than in developed countries (1). In 2022, the World Health Organization, through the International Agency for Research on Cancer, reported that in China, the incidence of breast cancer among cancers was as high as 7.4%, with a mortality rate of 2.9%. It ranks second among cancers in women, with patients comprising 14.9% of female cancer cases (2). According to the National Cancer Center (NCC) of China, in 2022, breast cancer ranked second among the most common cancers in Chinese women, with 357,200 new cases. It was the fifth leading cause of cancer-related deaths in women (3). It is evident that, over time, the burden of breast cancer in China has become increasingly severe.

Clinically, breast cancer treatment methods include surgery, radiotherapy, chemotherapy, endocrine therapy, and targeted therapy. However, these treatment methods can bring a series of side effects, such as pain, vomiting, and sleep disturbances. Due to the effects of breast cancer itself and various treatment-related side effects, CRF (cancer-related fatigue) has become one of the common burdens of breast cancer (4). In 2019, the U.S. Food and Drug Administration (FDA) stated that adverse drug reactions are a significant component of patient-centered health-related quality of life (HRQOL) and patient-reported outcomes (PRO) (5, 6). These adverse reactions not only increase the treatment burden on patients but also reduce treatment adherence and may even indirectly increase the mortality risk for breast cancer patients (7). Over the past decade, through the large-scale integration of genomic and transcriptomic data, as well as multidimensional combinations of single-cell and spatial technologies (8), we have gained a deeper understanding of the biological mechanisms of breast cancer. This has led to improved treatment strategies, increased patient survival rates, and a higher number of long-term survivors. However, the degree of fatigue experienced during and after treatment still far exceeds that of pain (9). About 33% of breast cancer patients will experience moderate to severe fatigue, which can persist for months or even years. Unlike ordinary fatigue, CRF is a physical, emotional, and cognitive exhaustion that cannot be relieved by rest. It can even develop into a chronic condition, affecting breast cancer patients’ ability to complete treatment, recover, and achieve a satisfactory quality of life and survival (10). Ultimately, it impacts their overall lifespan.

Therefore, preventing and effectively treating CRF remains a significant challenge. Historically, acupuncture has not been used to cure cancer itself, but to alleviate the side effects that arise during cancer treatment (11). A clinical practice guideline indicates that acupuncture has been proven effective in alleviating nausea, vomiting, pain, musculoskeletal disorders, hot flashes, fatigue, stress, anxiety, and sleep disturbances induced by chemotherapy (12). Clinical studies have also found that acupuncture can reduce CRF in breast cancer patients (13), improve sleep quality (14), alleviate anxiety and depression (15), relieve hot flashes (16), and enhance patients’ quality of life (13). At the same time, an increasing number of systematic reviews indicate that acupuncture has positive effects on a range of symptoms experienced by breast cancer survivors (17–20). In a 2017 consensus statement, the National Cancer Institute (NCI) mentioned that the current clinical applications of acupuncture in oncology primarily focus on single symptoms or conditions. Future research directions for acupuncture include studies on common symptom clusters, such as pain, sleep disturbances, fatigue, and psychological distress (21). ATAS is a new treatment method proposed by Professor Miansheng Zhu, based on the application of four traditional time acupuncture methods and the incorporation of European medical cultural concepts. It explores the clinical efficacy of ATAS for cancer symptom clusters.

### Objectives

In this study, we focus on survivors of postoperative chemotherapy for breast cancer, specifically reporting on the impact of ATAS on fatigue in these patients. The aim is to assist and guide breast cancer survivors suffering from fatigue and to explore whether ATAS is more effective in reducing CRF with fewer side effects.

## Methods

### Trial design

In September 2017, a project discussion meeting involving breast cancer experts from China and France was organized in Kunming, Yunnan Province, China. Subsequently, leveraging the resources of Yunnan Cancer Hospital and with French precision medicine platform experts as contributors, a methodological design was developed based on the clinical characteristics of ATAS. The clinical trial protocol was completed, covering statistical foundations, efficacy evaluation methods, and implementation processes. The complete date of the first trial registration for this experiment was 11/12/2017. The research was approved by the Ethics Committee of Yunnan Cancer Hospital, approval number YJZ201705; Chinese Clinical Trial Registration number ChiCTR-IPR-17013652.All eligible participants signed informed consent forms before enrollment, and the trial was reported according to the Consolidated Standards of Reporting Trials (CONSORT) guidelines (**Supplementary Material 1**).

### Participants

All diagnoses were confirmed through pathological histology, mammography, and other examinations, following the diagnostic criteria for breast cancer outlined in the *Guidelines and Norms for the Diagnosis and Treatment of Breast Cancer (2015 Edition)* by the Chinese Anti-Cancer Association. Inclusion criteria: 1. Meeting the diagnostic criteria for breast cancer, having undergone breast cancer surgery, and with no evidence of distant metastasis; 2. Meeting the criteria for adjuvant chemotherapy; 3. Patients aged between 18 and 60 years; 4. KPS score of ≥60. Exclusion criteria: 1. Known severe inflammation or metabolic disease; 2. Having received acupuncture treatment within the past 4 months; 3. Treated for cancer within five years prior to recruitment; 4. Mental disorders; 5. Patients with a fear of needles; 6. Skin disease at the acupuncture sites.

### Calculation of sample size

The sample size was estimated using a superiority trial design with a 1:1:1 group ratio, and the sample size estimation formula is as follows: $n=2[\frac{\left(\mu_{1-\alpha }+\mu_{1-\beta }\right)s}{\epsilon }{]}^{2}$ .

In the above formula: n represents the sample size required for each group, α represents Type I error, β represents Type II error, 1-β represents power, s is the standard deviation, μ is the population mean, μ_1_ -α and μ_1_ -_β_ represent the one-sided critical values corresponding to 1-α and 1-β, and ϵ is the effect size difference between the experimental group and the control group. Based on relevant literature and Professor Zhu Miansheng’s previous experience in treating CRF, the improvement in the Space-time Acupuncture Linggui Bafa group was 1.45 points higher than that in the control and sham acupuncture groups, i.e., ϵ = 1.45; the standard deviation for the three groups was assumed to be 2.0, i.e., s = 2.0. α = 0.025 (one-sided) and β = 0.2 were used. Substituting these parameters into the formula, 30 valid cases are required for each group. With a 1:1:1 parallel design for the three groups, a total of 90 valid cases need to be treated and followed up. Considering a dropout rate of no more than 20%, a total of 108 participants are required to be included.

### Randomization and blinding

After participants provided consent and signed the informed consent form, we used stratified block randomization for group assignment. The 108 enrolled patients were stratified into three groups based on age (18-30 years, 31-60 years). In the first step of grouping, the patients were divided into 12 groups, each consisting of 9 participants, for a total of 108 subjects. Next, randomization was performed. Participants were sequentially numbered based on their order of visits. Starting from a specific position on the random number table, consecutive random numbers were selected and assigned to the corresponding groups: numbers 1-3 for group A (ATAS group), numbers 4-6 for group B (sham acupuncture group), and numbers 7-9 for group C (waitlist control group). Each group consisted of 36 participants, maintaining a 1:1:1 ratio. Due to the specific nature of acupuncture procedures, our study employed a single-blind design. Only the acupuncturists and the research coordinators who had no contact with the patients were aware of the random group assignments.

### Recruitment and data collection

**Figure 1:**
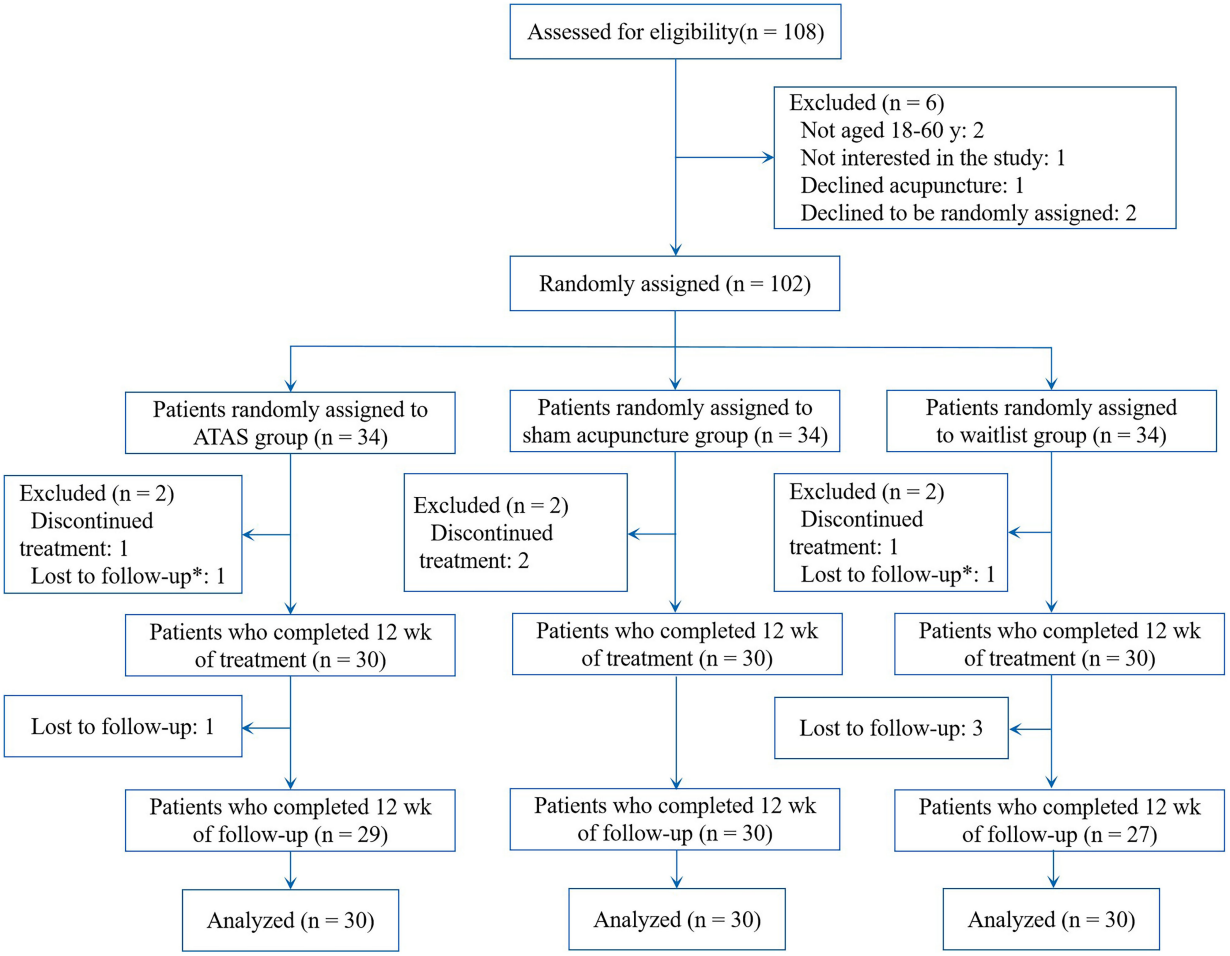
Enrolment, treatment, and follow-up.

This study recruited participants from the breast cancer ward at Yunnan Cancer Hospital between March 2018 and December 2019. Recruitment was primarily conducted by the doctors and nurses in the ward. We contacted interested participants by phone to screen for eligibility based on the inclusion criteria. Eligible participants were provided with detailed information about the study. They voluntarily chose to participate or decline and were informed that they had the right to withdraw at any time without any impact on their treatment (**Figure 1**).

### Intervention

#### Adjuvant chemotherapy

The three groups of patients used chemotherapy as follows: Epirubicin and Cyclophosphamide followed by Paclitaxel (Epirubicin 90 mg/m^2^ plus Cyclophosphamide 600 mg/m^2^, q3w x4, followed by Paclitaxel 175 mg/m^2^, q2w x4). Chemotherapy cycle: Chemotherapy was completed in one day. Each chemotherapy session was followed by a 3-week interval (21 days) before the next cycle, for a total of 4 cycles (12 weeks). Throughout the entire course of adjuvant chemotherapy, patients enrolled in this study—except for those in the waitlist control group—received one acupuncture treatment (ATAS or sham acupuncture) per week. In practice, if acupuncture and chemotherapy were scheduled on the same day, the acupuncture treatment was completed before the chemotherapy session. During weeks without chemotherapy, acupuncture was scheduled on the same weekday as in the chemotherapy weeks (e.g., if chemotherapy was on Friday, acupuncture was also scheduled for Friday during non-chemotherapy weeks).

#### ATAS

ATAS is a new acupuncture method that primarily consists of time acupoints and space acupoints. It employs specific implementation techniques and acupuncture sequences to achieve therapeutic effects for various diseases. ATAS consists of four systems: Space-time acupuncture Na Jia method, Space-time acupuncture Na Zi method, Space-time acupuncture Linggui Bafa, and Space-time acupuncture Feiteng Bafa (22). When applying ATAS, time acupoints are selected first, followed by the selection and manipulation of space acupoints.

This study focused on the use of the Space-time acupuncture Linggui Bafa. The time points mainly consist of the following four groups of acupoints: SP4 (Gongsun) and PC6 (Neiguan), LU7 (Lieque) and LI6 (Zhaohai), BL62 (Shenmai) and SI3 (Houxi), GB41 (Zulinqi) and TE5 (Waiguan). The group of acupoints should be selected based on the patient’s visit time or the different causes of fatigue, with all acupoints in each group used simultaneously. The first acupoint is the primary acupoint, and the second is the auxiliary acupoint. If the symptoms or lesions are not unilateral, during acupuncture, male patients should first needle the acupoints on the left side, while female patients should first needle the acupoints on the right side. When the symptoms are localized to the left or right side of the body, acupoints on the opposite side should be treated first. For example, in the case of left-sided breast cancer, the acupoints on the right side should be needled first, regardless of gender.

**Figure 2:**
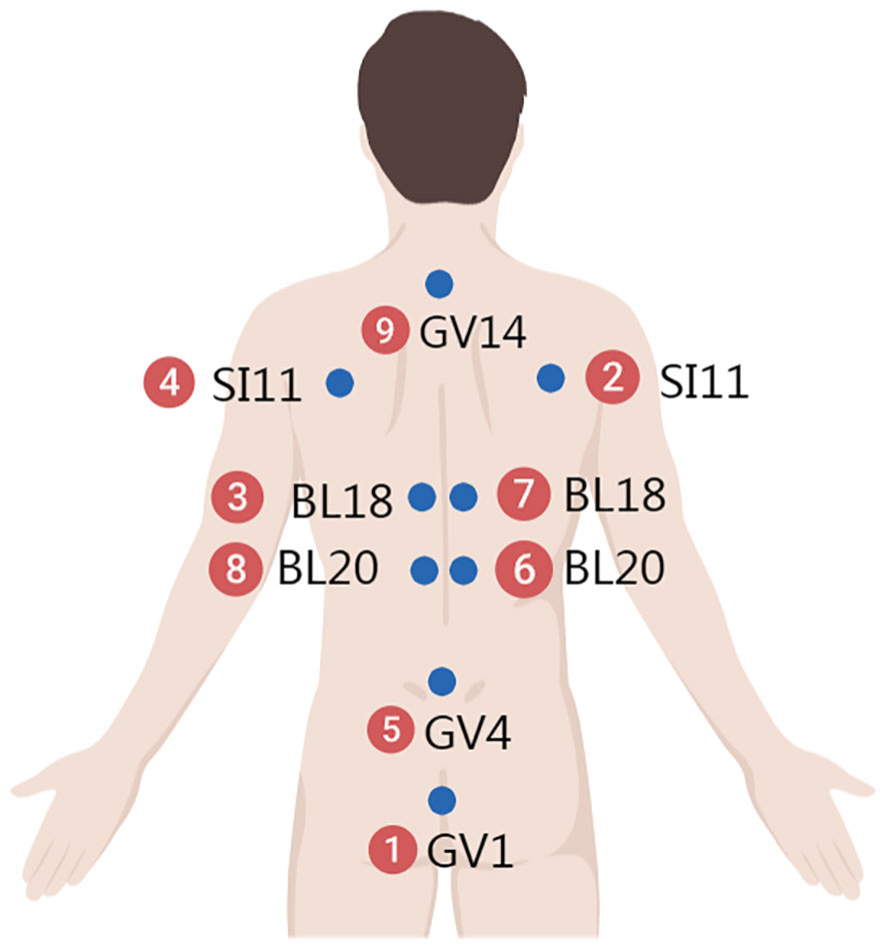
Neck and back.

**Figure 3:**
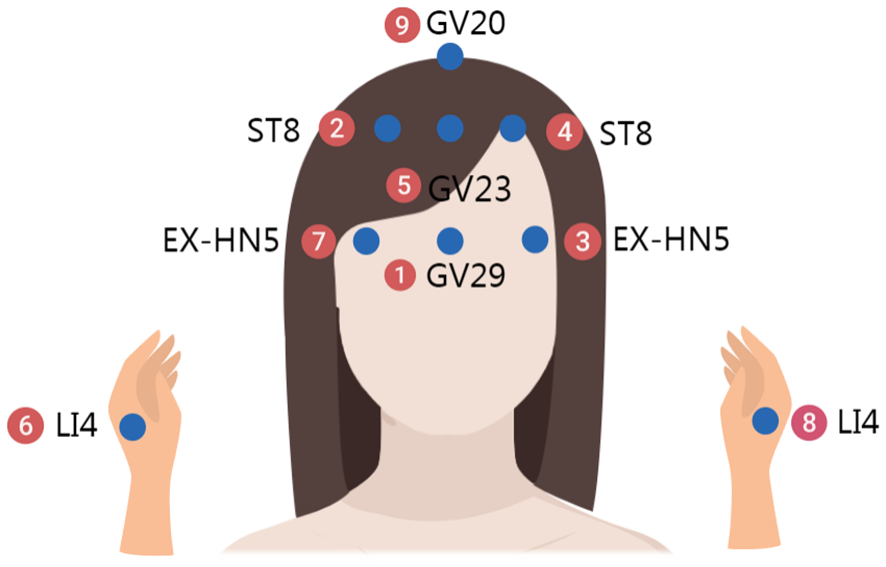
Head and hands.

**Figure 4:**
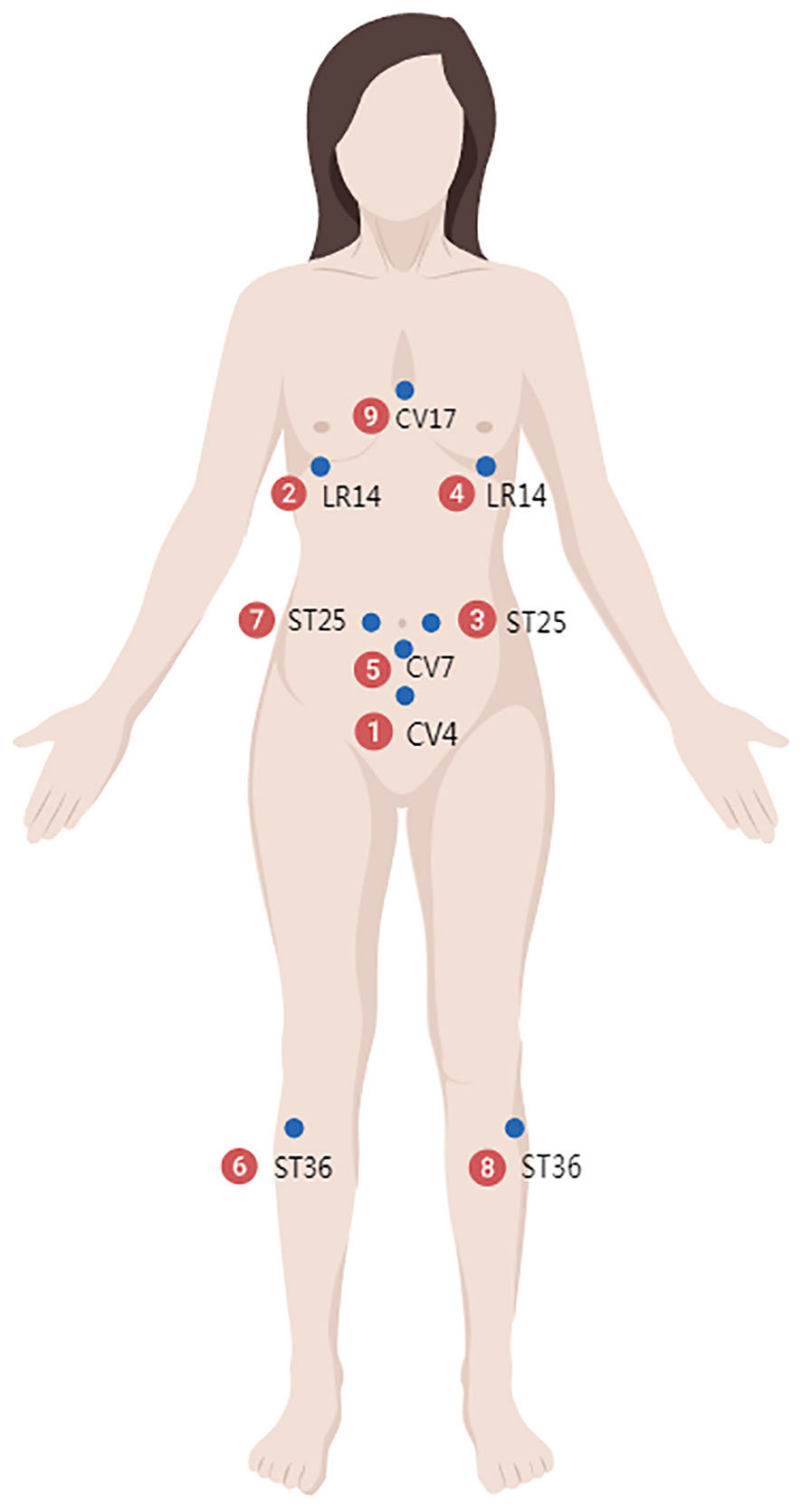
Chest, abdomen, and lower limbs.

**Figure 5:**
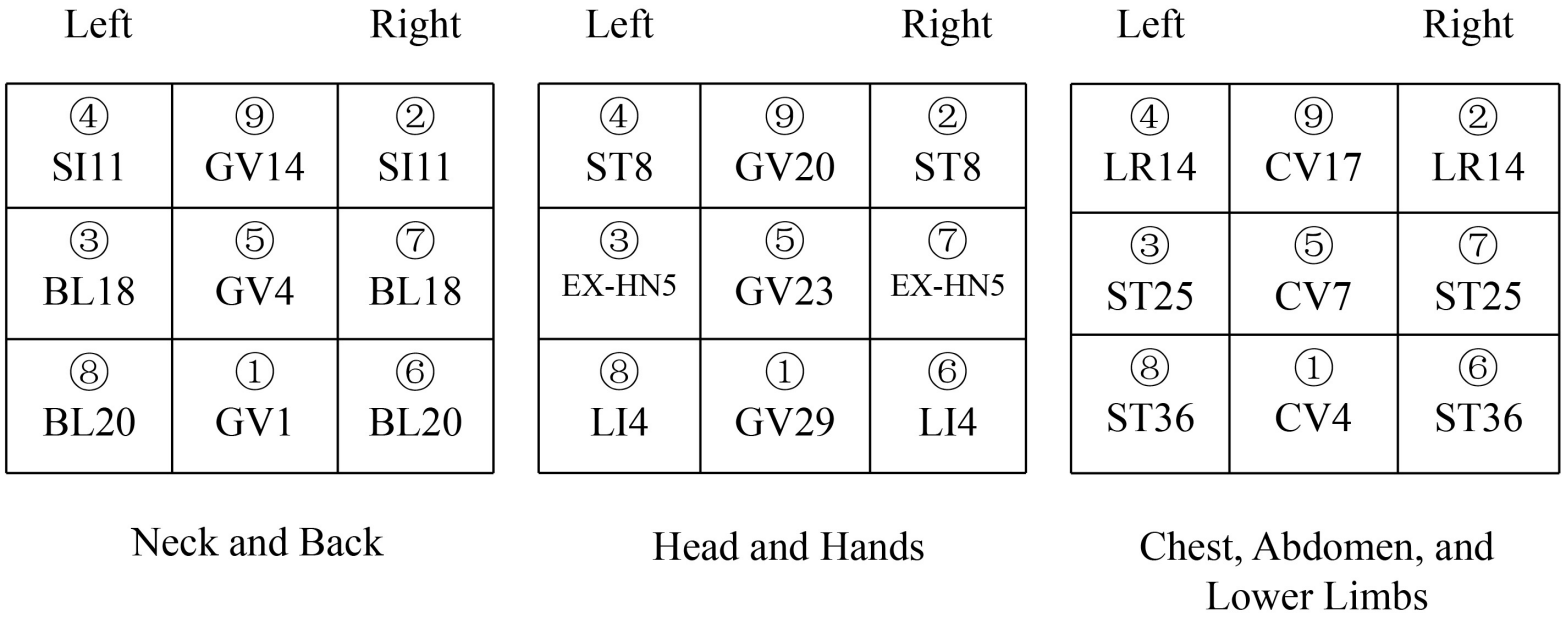
Needle puncture site and sequence in the ATAS group.

The space acupoints consist of three parts, distributed across the neck and back (**Figure 2**), the head and hands (**Figure 3**), and the chest, abdomen, and lower limbs (**Figure 4**). Each of these three areas consists of nine acupoints. During acupuncture, the first space acupoint area is selected based on time, and acupuncture is performed sequentially from point ① to point ⑨. The left and right in the picture is subject to the patient (**Figure 5**).

For example, on August 16, 2017, at 11:00, at Yunnan Provincial Cancer Hospital, the time acupoints were TE5 (Waiguan) and GB41 (Zulinqi), with TE5 (Waiguan) needled first, followed by GB41 (Zulinqi). If the patient is female and diagnosed with right-sided breast cancer, the acupuncture sequence is as follows: left TE5 (Waiguan), right TE5 (Waiguan), left GB41 (Zulinqi), and right GB41 (Zulinqi). The space acupoint TE5 (Waiguan) is located at position ③, so the space acupoints begin at ③ and proceed in the sequence: ③→④→⑤→⑥→⑦→⑧→⑨→①→② to complete all 9 acupoints. For example, for the neck and back acupoints, begin with left BL18, followed by left SI11, GV4, right BL20, right BL18, left BL20, GV14, GV1, and right SI11, with the same sequence applied to the other two areas.

The first acupuncture treatment is administered before the first chemotherapy session following surgery. Subsequent treatments are conducted once a week for a total of 12 sessions, with each session involving 30 minutes of needle retention. All practitioners hold a medical practitioner’s license and have more than five years of experience in acupuncture and massage therapy.

#### Sham acupuncture

**Figure 6:**
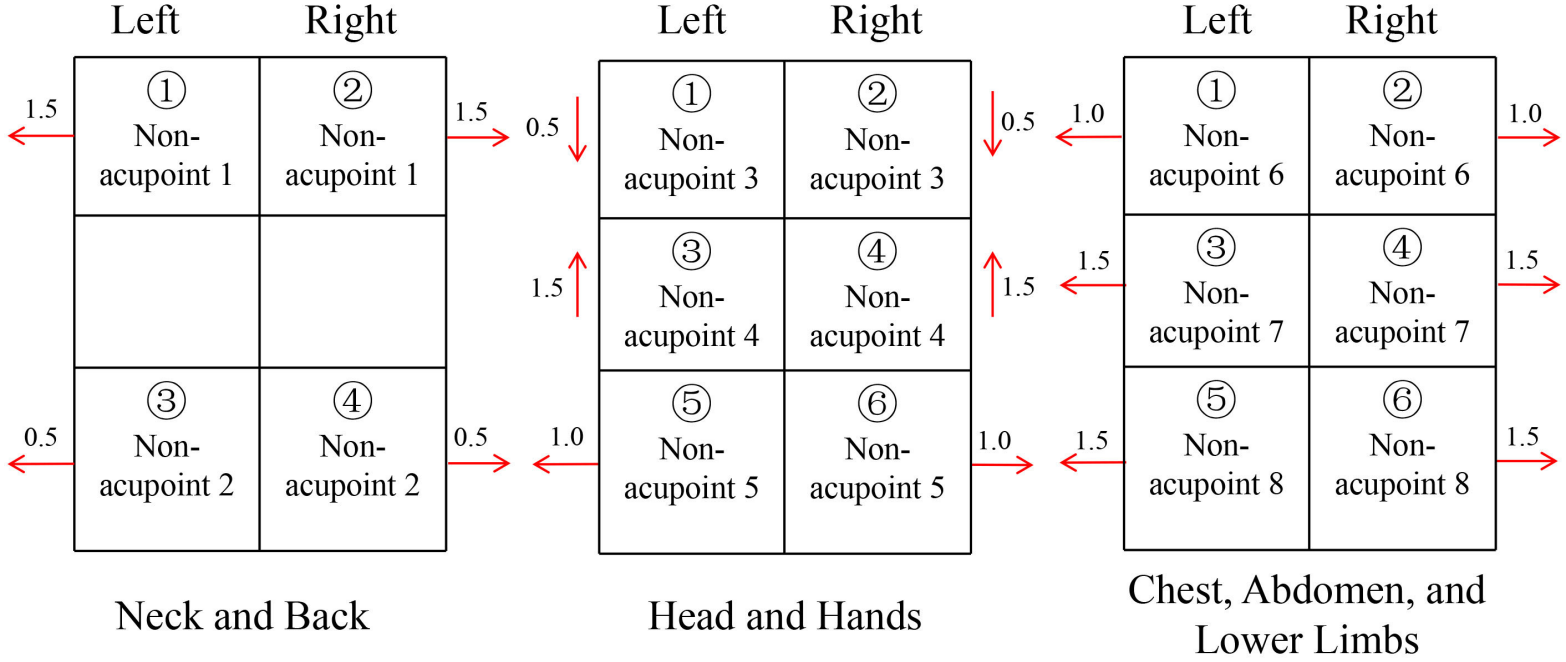
Needle puncture site and sequence in the Sham acupuncture group.

Patients in the sham acupuncture group will receive non-penetrating sham acupuncture at locations away from traditional acupoints. A literature review was conducted to exclude acupoints documented for treating CRF in breast cancer, and 16 non-acupoint stimulation points were selected. These points are not located on traditional acupuncture points but are positioned around the acupoints related to Space-time acupuncture Linggui Bafa. The positions of the stimulation points are described below, following the order of acupuncture. All stimulation points are needled with shallow insertion, just through the skin (1-4 mm), without any manipulation. The duration and frequency of treatments were the same as those in the ATAS group. (**Figure 6**).

**Supplementary Material 2** provides the specific locations of acupoints in the ATAS and sham acupuncture groups.

#### Waitlist control group

The waitlist control group was informed that they would only participate in adjuvant chemotherapy and not any acupuncture treatment. Treatments are: Epirubicin and Cyclophosphamide followed by Paclitaxel (Epirubicin 90 mg/m^2^ plus Cyclophosphamide 600 mg/m^2^, q3w x4, followed by Paclitaxel 175 mg/m^2^, q2w x4).

### Outcome measures

#### Primary outcome

Primary outcome: The Piper Fatigue Scale (PFS), revised by Piper et al. The scale consists of 24 items in total. The first two items assess the duration of fatigue, while the remaining 22 items are divided into four subjective dimensions: items 3 to 8 assess the behavioral dimension, items 9 to 13 assess the emotional dimension, items 14 to 18 assess the sensory dimension, and items 19 to 24 assess the cognitive dimension. The four dimensions assess the impact of fatigue on daily activities, emotions, and memory. All items are rated on a scale from 0 to 10, with 0 indicating the least severity and 10 indicating the highest severity.

#### Secondary outcomes

1. Insomnia Severity Index (ISI): This scale consists of 7 items, with higher scores indicating more severe insomnia. 2. Hospital Anxiety and Depression Scale (HADS): This scale is primarily used to screen for anxiety and depression in hospital patients. It consists of 14 items, with 7 items assessing depression (HADS-D) and 7 items assessing anxiety (HADS-A). 3. IL-2, IL-6, CD3^+^T, CD4^+^T: IL-2 and IL-6 were detected by chemiluminescent immunoassay (CLIA). CD3^+^T and CD4^+^T were detected by flow cytometry (FCM).

### Statistical analysis

Data analysis was conducted using SPSS 28.0 software. The data in this study are presented as “ $\bar{x}$ ± s”, and normality and homogeneity of variance tests were conducted. For comparisons between three groups that met the assumption of normal distribution, one-way ANOVA was used. If homogeneity of variance was assumed, the LSD method was applied; if homogeneity was not assumed, the Tamhane’s T2 method was used. Non-normally distributed data were analyzed using the rank-sum test. Categorical data were analyzed using the chi-square test. A *P*-value of < 0.05 was considered statistically significant.

## Results

### Participants

A total of 108 postoperative chemotherapy patients with breast cancer were screened for eligibility, and 102 qualified participants (94.4%) were randomly assigned. **Figure 1** shows the screening process and the reasons for exclusion. Thirty participants were assigned to receive ATAS, thirty to receive sham acupuncture, and another thirty to the waitlist control group. A total of 90 participants (100%) completed the intervention, and 86 participants (95.6%) completed the follow-up assessment.

Twelve participants (11.8%) withdrew from the study for various reasons: 4 from the ATAS group, 4 from the sham acupuncture group, and 4 from the waitlist group. The participants’ ages ranged from 18 to 60 years, and all were female. The average age in the ATAS group was 48.85 years [95% CI, 40.82 to 56.78], 49.6 years in the sham acupuncture group [95% CI, 40.16 to 59.04], and 48.03 years in the waitlist group [95% CI, 39.64 to 56.42]. **Table 1** shows the baseline characteristics of the three groups.

**Table 1:** Baseline characteristics of the participants.

Characteristic	ATAS (n=30)	Sham Acupuncture (n=30)	Waitlist Group (n=30)
Mean age (95% CI), year	48.80(40.82-56.78)	49.60(40.16-59.04)	48.03(39.64-56.42)
Mean BMI (95% CI), kg/m^2^	21.20(19.99-22.41)	22.50(21.45-23.55)	21.90(20.34-23.46)
Female sex, n (%)	30(100)	30(100)	30(100)
Education level, n (%)			
University	3(10)	2(7)	3(10)
High school	17(57)	15(50)	18(60)
Other	10(33)	13(43)	9(30)
Marital status, n (%)			
Married	25(83.3)	26(86.7)	28(93.3)
Other marital status	5(16.7)	4(13.3)	2(6.7)
Employment status, n (%)			
Employed	13(43.3)	10(33.3)	14(46.7)
Unemployed	7(23.3)	6(20)	8(26.7)
Retired	10(33.3)	14(46.7)	8(26.7)
Mean PFS Total (95% CI)†	5.78(4.90-6.66)	5.57(4.61-6.53)	5.68(5.09-10.77)
Mean ISI (95% CI)†	17.67(13.56-21.78)	17.17(13.85-20.46)	17.23(14.59-19.87)
Mean HADS (95% CI)			
HADS-A	11.97(6.87-17.07)	10.77(6.69-14.85)	9.70(5.79-13.61)
HADS-D	11.17(6.78-15.56)	10.00(6.49-13.51)	9.20(5.72-12.68)
IL-2	23.20(12.53-33.87)	24.43(13.71-35.15)	22.43(11.44-3.42)
IL-6	28.43 (9.88-46.98)	27.81(10.48-45.14)	28.00(8.94-47.06)
CD3^+^T	68.63(58.83-78.43)	74.46(64.36-84.56)	70.32(59.11-81.53)
CD4^+^T	36.84(28.25-45.43)	42.31(33.65-50.97)	39.77(31.02-48.52)

BMI, Body Mass Index; HADS, Hospital Anxiety and Depression Scale; IL-2, Interleukin-2; IL-6, Interleukin-2; ISI, Insomnia Severity Index; PFS, PiPer Fatigue Scale.

†Higher scores indicate worse symptoms.

### Primary outcome

In the ATAS group, the total PFS score after the intervention was 4.98 [95% CI, 3.96 to 6.00], showing a decrease of 0.8 points from the baseline (decreasing from 5.78 to 4.98, *P* < 0.05). The scores for each dimension after the intervention were as follows: Behavior Dimension, 5.10 [95% CI, 3.80 to 6.40], Affection Dimension, 5.57 [95% CI, 4.53 to 6.61], Perception Dimension, 5.07 [95% CI, 3.45 to 6.69] and Cognition Dimension, 4.30 [95% CI, 2.93 to 5.67]. All of these changes were statistically significant (*P* < 0.05). In the sham acupuncture group, the total PFS score after the intervention was 5.67 [95% CI, 4.90 to 6.44], showing an increase of 0.1 points from the baseline (*P* > 0.05). Additionally, the scores for the four dimensions before and after the intervention showed no statistically significant differences (*P* > 0.05). The Behavior Dimension is 5.87 [95% CI, 4.65 to 7.09], the Affection Dimension is 6.17 [95% CI, 5.15 to 7.19], the Perception Dimension is 5.73 [95% CI, 4.82 to 6.64], and the Cognition Dimension is 4.90 [95% CI, 3.71 to 6.09]. In the waitlist control group, the total PFS score increased from 5.68 [95% CI, 5.09 to 6.77] to 6.47 [95% CI, 5.93 to 7.01]. The scores for all four dimensions increased, with the magnitude of the increase greater than that in the sham acupuncture group. The Behavior Dimension is 6.60 [95% CI, 5.71 to 7.49], the Affection Dimension is 6.97 [95% CI, 5.87 to 8.07], the Perception Dimension is 6.37 [95% CI, 5.48 to 7.26], and the Cognition Dimension is 5.93 [95% CI, 4.73 to 7.13]. After completing the treatment, statistical analysis was conducted for the three groups. The ATAS group showed significantly better results in total PFS scores, as well as in the Behavior Dimension, Affection Dimension, Perception Dimension, and Cognition Dimension, with all differences statistically significant (*P* < 0.05) (**Table 2**).

**Table 2:** Outcomes.

Outcome	ATAS (n=30)	Sham Acupuncture (n=30)	Waitlist Group (n=30)	P value
PFS Total (95% CI)	4.98(3.96-6.00) ^a^	5.67(4.90-6.44)	6.47(5.93-7.01)	< 0.01^b^
Behavior Dimension	5.10(3.80-6.40) ^a^	5.87(4.65-7.09)	6.60(5.71-7.49)	< 0.01 ^b^
Affection Dimension	5.57(4.53-6.61) ^a^	6.17(5.15-7.19)	6.97(5.87-8.07)	< 0.01 ^b^
Perception Dimension	5.07(3.45-6.69) ^a^	5.73(4.82-6.64)	6.37(5.48-7.26)	< 0.01 ^b^
Cognition Dimension	4.30(2.93-5.67) ^a^	4.90(3.71-6.09)	5.93(4.73-7.13)	< 0.01 ^b^
ISI (95% CI)	15.17(12.28-18.06) ^a^	16.70(13.17-20.23)	18.30(15.30-21.30)	< 0.01 ^b^
HADS (95% CI)				
HADS-A	8.63(5.18-12.08) ^a^	10.43(6.85-14.01)	12.37(8.94-15.80)	< 0.01 ^b^
HADS-D	7.80(4.73-10.87) ^a^	8.97(6.20-11.74) ^a^	10.93(7.49-14.37)	< 0.01 ^b^
IL-2	20.18(11.51-28.85) ^a^	23.52(13.0-34.03)	27.55(14.92-40.18)	0.033 ^b^
IL-6	24.56(7.57-41.55) ^a^	27.72(11.45-43.99)	36.08(16.21-55.95)	0.039 ^b^
CD3^+^T	79.03(68.56-89.50) ^a^	73.78(63.52-84.31)	72.47(61.79-83.15)	0.044 ^b^
CD4^+^T	42.89(35.14-50.64) ^a^	41.11(32.49-49.73)	36.51(27.21-45.81)	0.015 ^b^

HADS, Hospital Anxiety and Depression Scale; IL-2, Interleukin-2; IL-6, Interleukin-2; ISI, Insomnia Severity Index; PFS, PiPer Fatigue Scale.

^a^Compared to baseline, *P*<0.05.

^b^Compared with the three groups, *P*<0.05.

### Secondary outcomes

In the ATAS group, the ISI score after the intervention was 15.17 [95% CI, 12.28 to 18.06], showing a decrease of 2.5 points from the baseline (from 17.67 to 15.17, *P* < 0.05). In the sham acupuncture group, the ISI score after the intervention was 16.70 [95% CI, 13.17 to 20.23], showing a decrease of 0.47 points from the baseline (from 17.17 to 16.70, *P* > 0.05). In the waitlist control group, the ISI score after the intervention was 18.30 [95% CI, 15.30 to 21.30], showing an increase of 1.07 points from the baseline (from 17.23 to 18.30, *P* > 0.05). After treatment, the difference in ISI scores between the ATAS group and the other two groups was statistically significant (*P* < 0.05).

In terms of HADS, the ATAS group showed a decrease in HADS-A scores from 11.97 [95% CI, 6.87 to 17.07] to 8.63 [95% CI, 5.18 to 12.08] (*P* < 0.05) and a decrease in HADS-D scores from 11.11 [95% CI, 6.78 to 15.56] to 7.80 [95% CI, 4.73 to 10.87] (*P* < 0.05). In the sham acupuncture group, the HADS-A score decreased from 10.77 [95% CI, 6.69 to 14.85] to 10.43 [95% CI, 6.85 to 14.01], with a decrease of 0.34 (*P* > 0.05). The HADS-D score decreased from 10 [95% CI, 6.49 to 13.51] to 8.97 [95% CI, 6.20 to 11.74] (*P* < 0.05). In the waitlist control group, the scores increased: HADS-A rose from 9.7 [95% CI, 5.79 to 13.61] to 12.37 [95% CI, 8.94 to 15.80], and HADS-D increased from 9.20 [95% CI, 5.72 to 12.68] to 10.93 [95% CI, 7.49 to 14.37]. After treatment, the ATAS group showed significant advantages in HADS scores compared to the other two groups, with statistically significant differences (*P* < 0.05).

In the ATAS group, after the intervention, IL-2 and IL-6 scores decreased by 3.02 points (a decrease from 23.20 to 20.18, *P* < 0.05) and 3.87 points (a decrease from 28.43 to 24.56, *P* < 0.05), respectively. At the same time, CD3^+^T and CD4^+^T levels increased. CD3^+^T increased from 68.63 [95% CI, 58.83 to 78.43] to 79.03 [95% CI, 68.56 to 89.50], and CD4^+^T increased from 36.84 [95% CI, 28.25 to 45.43] to 42.89 [95% CI, 35.14 to 50.64]. In the sham acupuncture group, after the intervention, IL-2 and IL-6 levels decreased by only 0.91 points (a decrease from 24.43 to 23.52, *P* > 0.05) and 0.09 points (a decrease from 27.81 to 27.72, *P* > 0.05), respectively. CD3^+^T and CD4^+^T levels decreased compared to before the intervention. In the waitlist control group, IL-2 and IL-6 levels showed an increasing trend compared to before the intervention. IL-2 increased from 22.43 [95% CI, 11.44 to 33.42] to 27.55 [95% CI, 14.92 to 40.18], and IL-6 increased from 28.00 [95% CI, 8.94 to 47.06] to 36.08 [95% CI, 16.21 to 55.95]. CD3^+^T increased from 70.32 [95% CI, 59.11 to 81.53] to 72.47 [95% CI, 61.79 to 83.15], while CD4^+^T decreased by 3.26 points. After treatment, the differences in IL-2, IL-6, CD3^+^T, and CD4^+^T between the ATAS group and the other two groups were statistically significant (*P* < 0.05).

### Adverse events

Seven participants in the ATAS group and the sham acupuncture group reported a total of nine adverse events. These adverse events included hematoma, bleeding, or pain around the acupuncture sites. No adverse events were reported in the waitlist control group (**Table 3**).

**Table 3:** Adverse events related to acupuncture*.

Adverse Event	Acupuncture (n = 60)†	
	Participants, n (%)	Events, n
Total	7 (11.7)	9
Hematoma around the site of needling	5 (8.3)	7
Needle left in participant	0 (0.0)	0
Fainted during acupuncture	0 (0.0)	0
Severe sharp pain during acupuncture	0 (0.0)	0
Sharp pain lasting >1 h	1 (1.7)	1
Bleeding around the site of needling	1 (1.7)	1
Infection around the site of needling	0 (0.0)	0
Other discomforts after acupuncture	0 (0.0)	0

* No adverse events occurred in the waitlist control group (n=30).

† All adverse events were mild and transient, and all participants recovered without further medical intervention. No patient withdrew from the study due to adverse events.

## Discussion

### Principal findings

Our randomized controlled trial indicates that, compared to sham acupuncture and waitlist control, ATAS intervention significantly alleviated fatigue in postoperative breast cancer patients. It also resulted in greater reductions in secondary outcome measures such as insomnia, anxiety, and depression compared to the sham acupuncture group. Additionally, ATAS lowered levels of inflammatory cytokines, improved the inflammatory status of patients, and had a positive effect on their quality of life. In the ATAS group and the sham acupuncture group, a total of nine adverse events occurred, with an incidence rate of 11.7%. All adverse reactions were mild and transient, requiring no medical intervention. The occurrence of adverse events might have been due to a failure to apply pressure immediately after needle removal. This should be addressed in future clinical practice.

In terms of outcome measures, our study focused on patients’ subjective experiences, such as fatigue, insomnia, anxiety, and depression. Emphasizing patients’ subjective perceptions, symptom improvement, satisfaction with disease diagnosis and treatment, and quality of life plays an irreplaceable role in evaluating clinical efficacy outcomes (23) Adjuvant treatments for breast cancer patients primarily include radiotherapy, chemotherapy, and endocrine therapy. Clinical studies on patients receiving chemotherapy have shown that 25% to 50% of these patients may develop peripheral neuropathy (24). Patients with stage I-III breast cancer experience poorer sleep, increased fatigue, and more severe depressive symptoms during chemotherapy (25, 26). Reports from breast cancer survivors indicate that most patients worry about disease recurrence, struggle to return to work, experience strained family relationships, and face psychological distress related to their social roles. As a result, after completing treatment, patients may experience varying degrees of pain, fatigue, sleep disturbances, and depression (27). These symptom clusters are persistent (28, 29) and significantly associated with multiple dimensions of quality of life (30).

Professor Zhu Miansheng is the founder of ATAS and a recipient of the French Knight of the Legion of Honor, First Class. Linggui Bafa is known as the “Qi Jing Na Gua” method in ancient China. A special structure is formed based on the Eight Trigrams and Luo Shu. Professor Zhu’s previous clinical studies revealed that Space-time acupuncture Linggui Bafa not only improves the symptoms of perimenopausal syndrome but also lowers serum FSH levels and raises E2 levels (31). It can further reduce the frequency of nighttime urination in elderly individuals, extend sleep duration, and enhance the overall quality of life for patients (32). The space acupoints of Space-time acupuncture Linggui Bafa consist of three groups of acupoints. In each group, the first acupuncture point corresponds to the acupoint associated with the time, and then, after combining with the Eight Trigrams, the other space acupoints are needled in sequence. Among the three groups of acupoints, the middle acupoints belong to the GV and CV. Taoism in China believes that this is a channel where energy converges. Therefore, we selected the acupoints of GV and CV, which reflect the characteristic of spatial acupoints gathering energy. SI11, CV17, ST25, BL18, BL20, and ST36 are commonly used and important acupoints for treating breast diseases. The space acupoints, as a whole, represent the three groups of acupoints as carriers and channels for the communication of the energy of heaven and earth.

ATAS is an innovation based on traditional acupuncture that combines time acupoints and space acupoints into a new acupuncture method (33). With the development of society and changes in people’s lifestyles, the spectrum of modern diseases has also undergone significant changes. Human diseases have shifted from primarily external invasive diseases to predominantly chronic and psychosomatic diseases. This phenomenon has become a major challenge for doctors. In clinical treatment, it is necessary to address the causes of fatigue in patients, tailoring the treatment according to the time, location, and individual characteristics. When selecting time acupoints in ATAS, five main methods are used: based on the time of psychological trauma, the time of accidents or natural disasters, the onset time of treatment-induced adverse reactions, diseases with specific onset times, and the patient’s birth time (34). The etiology and pathogenesis of CRF in breast cancer are complex, involving factors such as the cancer itself, surgery, and psychological trauma. In the treatment using the ATAS method, all these factors are taken into consideration. For example, if fatigue is significantly worsened after surgery, the time of the surgery would be selected as the time acupoint. Space acupoints are always used after time acupoints, following a specific sequence, and integrating the time points and space points into a cohesive system (35). This approach can address the disease itself while also having a positive effect on symptoms induced by the treatment. Additionally, in the ATAS method, the combination of time acupoints and space acupoints fully embodies a personalized treatment plan that considers the specific timing and individual needs of each patient. This trial achieved a close integration of traditional Chinese medicine’s individualized treatment approach with the standardized requirements of clinical trials, embodying the essence of acupuncture’s original principles. We hope that this clinical trial research will provide a reference design for traditional Chinese medicine clinical trials, offer clinicians better treatment options for breast cancer CRF, and enable breast cancer CRF patients to have more choices in their treatment.

Our study found that the total PFS score in the ATAS group decreased by 0.8 points after the intervention. The etiology of cancer-related fatigue may be related to immune-inflammatory responses (36). Di Meglio (37) found that high levels of IL-6 and IL-2 are associated with CRF. Cohen (38) found that IL-6 and IL-8 play a modulatory role in the relationship between physical activity and fatigue. Inflammatory cytokines from the peripheral system can transmit signals to the brain via the vagus nerve or endocrine system (39), triggering the further release of inflammatory mediators, leading to fatigue, cognitive dysfunction, or sleep disturbances, among other behavioral changes, in an attempt to restore homeostasis (40). At the same time, tissue damage caused by tumor treatments such as surgery, radiotherapy, and chemotherapy also triggers systemic inflammation, and psychological stress related to cancer diagnosis or treatment is one of the factors contributing to the imbalance of inflammatory factors (41). Sleep disorders are often observed before cancer treatment, and it has been confirmed that they coexist with other conditions such as anxiety and depression. Different breast cancer treatment methods increase the risk of sleep disorders, which, in turn, adversely affect the efficacy of cancer treatment (42). Cellular immunity is an essential component of the immune system, with T cells playing a central role in defending against pathogen invasion, eliminating cancer cells, preventing autoimmune diseases induced by pathogens and the environment, and limiting chronic inflammation, thereby maintaining immune homeostasis (43, 44). Research has shown that in breast cancer survivors with persistent fatigue, T lymphocyte levels are elevated, particularly CD4^+^ T cells (45). CD4^+^ T cells and CD8^+^ T cells interact and exert both positive and negative regulatory effects on immune responses, thereby jointly regulating immune homeostasis. Acupuncture also has a bidirectional regulatory effect, which may be related to the mechanism through which acupuncture modulates immune function.

Considering the relationship between inflammation and immune suppression, anti-inflammatory treatments and the restoration of host immunity may be effective strategies for cancer treatment (46). In recent years, numerous studies have investigated the mechanism of acupuncture’s anti-inflammatory effects (47, 48), which can be both local and systemic. The anti-inflammatory effect of acupuncture is primarily achieved in two ways: one through sensory stimulation from acupuncture to the central nervous system, stimulating the HPA axis, sympathetic, or vagal nerve pathways to regulate immune function. The other is through acupuncture as a minimally invasive stimulus to the body surface, inducing or enhancing the body’s own inflammatory reflex (49). Previous studies have shown that electroacupuncture is effective in treating various diseases involving excessive inflammatory responses (50, 51). A meta-analysis showed (52) that the acupuncture treatment group exhibited improved immune function, with increases in CD3 cells, CD4 cells, and the CD4/CD8 ratio, while IL-1, IL-4, IL-6, and CRP levels of inflammatory markers decreased. Acupuncture may improve immune outcomes and reduce inflammation during cancer treatment. A clinical cancer study found that, in the acupuncture group, CD3^+^ T and CD4^+^ T cell levels increased, while IL-6 and CRP levels decreased (53). A systematic review of 28 clinical studies showed that acupuncture can significantly reduce clinical scores of depression and anxiety in cancer patients (54), and the American Society of Integrative Oncology also recommends acupuncture for treating breast cancer-related negative emotions in its clinical guidelines (12). Regarding immune system regulation under emotional disturbances, studies suggest that electroacupuncture can reduce the elevation of inflammatory factors in the serum of animals subjected to postoperative stress or chronic adolescent restraint stress models (55, 56). Therefore, acupuncture may regulate immune responses by alleviating emotional disturbances in stressful conditions, and this immune modulation could be one of the potential mechanisms by which acupuncture contributes to cancer prevention and treatment in the future. Thus, selecting acupuncture for patients after breast cancer surgery can regulate immune function while also positively affecting accompanying symptoms. This study found that, in the ATAS group, the levels of CD3^+^ T lymphocytes and CD4^+^ T lymphocytes increased throughout the study, while IL-2 and IL-6 levels decreased. This indicates that ATAS positively affects the chronic inflammatory state in breast cancer patients after chemotherapy and enhances immune function in breast cancer CRF patients.

### Research implications

The limitations of this study include a small sample size and a short observation period, with effects observed only after one chemotherapy cycle post-surgery. The results related to quality of life, survival time, and mortality of breast cancer patients after treatment could not be immediately presented. In the next stage of the study, we plan to increase the sample size, conduct follow-up tracking, and perform further analysis. In the later stages of the study, due to the specificity of acupuncture procedures, the acupuncturist could not be blinded, which limits the single-blind design. Therefore, we blinded the outcome assessors and data analysts, who were not involved in the treatment procedures. The study reported only mild adverse events and lacked long-term safety assessments. In future studies, regular monitoring of patient fatigue, sleep disorders, anxiety, and depression should be conducted to assess whether they worsen. Additionally, imaging tests and estrogen levels should be monitored for abnormalities, and attention should be paid to the occurrence of cardiovascular diseases, fractures, and peripheral neuropathy.

Currently, the application of acupuncture in oncology treatment is still in its early stages, primarily used to manage adverse reactions induced by comprehensive treatments like radiotherapy and chemotherapy. Therefore, future research should not be limited to treating symptoms caused by tumors. More convincing research is needed to demonstrate the inhibitory effects of acupuncture on tumors, thereby fully leveraging the potential advantages of acupuncture in cancer treatment research.

## Conclusions

Our preliminary findings indicate that ATAS effectively improves fatigue in postoperative chemotherapy patients with breast cancer. It also has positive effects on sleep, anxiety, depression, and inflammatory factors. These results suggest that ATAS intervention may be an effective method for alleviating fatigue in breast cancer patients.

## Data Availability

The raw data supporting the conclusions of this article will be made available by the authors, without undue reservation.

## References

[1] Sung H Ferlay J Siegel RL Laversanne M Soerjomataram I Jemal A. Global cancer statistics 2020: GLOBOCAN estimates of incidence and mortality worldwide for 36 cancers in 185 countries. CA Cancer J Clin. (2021) 71:209–49. doi: 10.3322/caac.21660 33538338

[2] Population fact sheets. The international agency for research on cancer. Available online at: https://gco.iarc.fr/today/data/factsheets/populations/160-China-factsheets (Accessed April 5, 2021).

[3] Han B Zheng R Zeng H Wang S Sun K Chen R. Cancer incidence and mortality in China, 2022. J Natl Cancer Cent. (2024) 4:47–53. doi: 10.1016/j.jncc.2024.01.006 39036382 PMC11256708

[4] Devoogdt N De Groef A. Physiotherapy management of breast cancer treatment-related sequelae. J Physiother. (2024) 70:90–105. doi: 10.1016/j.jphys.2024.02.020 38519340

[5] Fiero MH Roydhouse JK Vallejo J King-Kallimanis BL Kluetz PG Sridhara R. US Food and Drug Administration review of statistical analysis of patient-reported outcomes in lung cancer clinical trials approved between January, 2008, and December, 2017. Lancet Oncol. (2019) 20:e582–9. doi: 10.1016/S1470-2045(19)30335-3 31579004

[6] Kluetz PG Slagle A Papadopoulos EJ Johnson LL Donoghue M Kwitkowski VE. Focusing on core patient-reported outcomes in cancer clinical trials: symptomatic adverse events, physical function, and disease-related symptoms. Clin Cancer Res. (2016) 22:1553–8. doi: 10.1158/1078-0432 26758559

[7] Makubate B Donnan PT Dewar JA Thompson AM McCowan C. Cohort study of adherence to adjuvant endocrine therapy, breast cancer recurrence and mortality. Br J Cancer. (2013) 108:1515–24. doi: 10.1038/bjc.2013.116 PMC3629427 23519057

[8] Nolan E Lindeman GJ Visvader JE. Deciphering breast cancer: from biology to the clinic. Cell. (2023) 186:1708–28. doi: 10.1016/j.cell.2023.01.040 36931265

[9] Cleeland CS Zhao F Chang VT Sloan JA O’Mara AM Gilman PB. The symptom burden of cancer: Evidence for a core set of cancer-related and treatment-related symptoms from the Eastern Cooperative Oncology Group Symptom Outcomes and Practice Patterns study. Cancer. (2013) 119:4333–40. doi: 10.1002/cncr.28376 PMC3860266 24114037

[10] Lahart IM Metsios GS Nevill AM Carmichael AR. Physical activity for women with breast cancer after adjuvant therapy. Cochrane Database Syst Rev. (2018) 1:CD011292. doi: 10.1002/14651858 29376559 PMC6491330

[11] Birch S Lee MS Alraek T Kim TH. Evidence, safety and recommendations for when to use acupuncture for treating cancer related symptoms: a narrative review. Integr Med Res. (2019) 8:160–6. doi: 10.1016/j.imr.2019.05.002 PMC6600712 31304088

[12] Greenlee H DuPont-Reyes MJ Balneaves LG Carlson LE Cohen MR Deng G. Clinical practice guidelines on the evidence-based use of integrative therapies during and after breast cancer treatment. CA Cancer J Clin. (2017) 67:194–232. doi: 10.3322/caac.21397 28436999 PMC5892208

[13] Molassiotis A Bardy J Finnegan-John J Mackereth P Ryder DW Filshie J. Acupuncture for cancer-related fatigue in patients with breast cancer: a pragmatic randomized controlled trial. J Clin Oncol. (2012) 30:4470–6. doi: 10.1200/JCO.2012.41.6222 23109700

[14] Garland SN Xie SX DuHamel K Bao T Li Q Barg FK. Acupuncture versus cognitive behavioral therapy for insomnia in cancer survivors: A randomized clinical trial. J Natl Cancer Inst. (2019) 111:1323–31. doi: 10.1093/jnci/djz050 PMC6910189 31081899

[15] Walker EM Rodriguez AI Kohn B Ball RM Pegg J Pocock JR. Acupuncture versus venlafaxine for the management of vasomotor symptoms in patients with hormone receptor-positive breast cancer: a randomized controlled trial. J Clin Oncol. (2010) 28:634–40. doi: 10.1200/JCO.2009.23.5150 20038728

[16] Lesi G Razzini G Musti MA Stivanello E Petrucci C Benedetti B. Acupuncture as an integrative approach for the treatment of hot flashes in women with breast cancer: A prospective multicenter randomized controlled trial (AcCliMaT). J Clin Oncol. (2016) 34:1795–802. doi: 10.1200/JCO.2015.63.2893 27022113

[17] Choi TY Ang L Jun JH Alraek T Birch S Lu W. Acupuncture for managing cancer-related fatigue in breast cancer patients: A systematic review and meta-analysis. Cancers (Basel). (2022) 14:4419. doi: 10.3390/cancers14184419 36139579 PMC9496910

[18] Li H Schlaeger JM Jang MK Lin Y Park C Liu T. Acupuncture improves multiple treatment-related symptoms in breast cancer survivors: A systematic review and meta-analysis. J Altern Complement Med. (2021) 27:1084–97. doi: 10.1089/acm.2021.0133 PMC8713255 34449251

[19] Zhang Y Sun Y Li D Liu X Fang C Yang C. Acupuncture for breast cancer: A systematic review and meta-analysis of patient-reported outcomes. Front Oncol. (2021) 11:646315. doi: 10.3389/fonc.2021.646315 34178633 PMC8222976

[20] Yuanqing P Yong T Haiqian L Gen C Shen X Dong J. Acupuncture for hormone therapy-related side effects in breast cancer patients: A GRADE-assessed systematic review and updated meta-analysis. Integr Cancer Ther. (2020) 19:1534735420940394. doi: 10.1177/1534735420940394 32718258 PMC7388099

[21] Zia FZ Olaku O Bao T Berger A Deng G Fan AY. The national cancer institute’s conference on acupuncture for symptom management in oncology: state of the science, evidence, and research gaps. J Natl Cancer Inst Monogr. (2017) 2017:lgx005. doi: 10.1093/jncimonographs/lgx005 29140486 PMC6061411

[22] Wang YH Wang LL Liu LX Zheng Z Zhu MS Wang ZH. Analysis of space-time acupuncture program of Linggui Bafa. China J Traditional Chin Med Pharm. (2021) 36:2756–9.

[23] Cui X Zhao TY Zhao MD Ren QQ Sang JL Guo Y. Acupuncture intervention for symptom clusters of breast cancer patients discussion on scheme ideas. J Tianjin Univ Traditional Chin Med. (2024) 43:247–52.

[24] Golan-Vered Y Pud D. Chemotherapy-induced neuropathic pain and its relation to cluster symptoms in breast cancer patients treated with paclitaxel. Pain Pract. (2013) 13:46–52. doi: 10.1111/j.1533-2500.2012.00554.x 22533683

[25] Liu L Fiorentino L Natarajan L Parker BA Mills PJ Sadler GR. Pre-treatment symptom cluster in breast cancer patients is associated with worse sleep, fatigue and depression during chemotherapy. Psychooncology. (2009) 18:187–94. doi: 10.1002/pon.1412 PMC2762479 18677716

[26] Gwede CK Small BJ Munster PN Andrykowski MA Jacobsen PB. Exploring the differential experience of breast cancer treatment-related symptoms: a cluster analytic approach. Support Care Cancer. (2008) 16:925–33. doi: 10.1007/s00520-007-0364-2 PMC2892385 18043948

[27] Andreu Y Galdón MJ Durá E Martínez P Pérez S Murgui S. A longitudinal study of psychosocial distress in breast cancer: prevalence and risk factors. Psychol Health. (2012) 27:72–87. doi: 10.1080/08870446.2010.542814 21678180

[28] Li H Sereika SM Marsland AL Conley YP Bender CM. Symptom clusters in women with breast cancer during the first 18 months of adjuvant therapy. J Pain Symptom Manage. (2020) 59:233–41. doi: 10.1016/j.jpainsymman.2019.10.002 31610271

[29] Li H Sereika SM Marsland AL Conley YP Bender CM. Impact of chemotherapy on symptoms and symptom clusters in postmenopausal women with breast cancer prior to aromatase inhibitor therapy. J Clin Nurs. (2019) 28:4560–71. doi: 10.1111/jocn.15047 31469461

[30] Roiland RA Heidrich SM. Symptom clusters and quality of life in older adult breast cancer survivors. Oncol Nurs Forum. (2011) 38:672–80. doi: 10.1188/11.ONF.672-680 PMC3711487 22037330

[31] Li CL Tian CY Guan J Deng YP Zhang F Liao X. To study the effect of Space-time acupuncture Linggui Bafa on perimenopausal syndrome and its effect on serum FSH and E2 levels. Chin Acupuncture Moxibustion. (2019) 39:1214–6. doi: 10.13703/j.0255-2930.2019.11.020

[32] Han LH Liu L Song M Zhang HJ Pan XJ. Clinical observation on the effect of Space-time acupuncture Linggui Bafa on senile nocturia. Yunnan J Traditional Chin Med Materia Med. (2022) 43:39–42. doi: 10.16254/j.cnki.53-1120/r.2022.04.007

[33] Zuo Z Zhu MS. An exploration of zhu miansheng’s ATAS. China J Traditional Chin Med Pharm. (2019) 34:924–6.

[34] Zuo Z Zhu MS Chen CX Yuan K Liu LX. Prof. Zhu miansheng’s theoretical basis of ATAS. Lishizhen Med Materia Med Res. (2018) 29:1740–3.

[35] Liu LX Wang ZH Bao XY Wang LL Zhu MS. Zhu miansheng’s essentials of ATAS. China J Traditional Chin Med Pharm. (2020) 35:84–8.

[36] Thong MSY van Noorden CJF Steindorf K Arndt V. Cancer-related fatigue: causes and current treatment options. Curr Treat Options Oncol. (2020) 21:17. doi: 10.1007/s11864-020-0707-5 32025928 PMC8660748

[37] Di Meglio A Havas J Pagliuca M Franzoi MA Soldato D Chiodi CK. A bio-behavioral model of systemic inflammation at breast cancer diagnosis and fatigue of clinical importance 2 years later. Ann Oncol. (2024) 35:1048–60. doi: 10.1016/j.annonc.2024.07.728 39098454

[38] Cohen M Levkovich I Katz R Fried G Pollack S. Low physical activity, fatigue and depression in breast cancer survivors: Moderation by levels of IL-6 and IL-8. Int J Psychophysiol. (2020) 158:96–102. doi: 10.1016/j.ijpsycho.2020.09.011 33080293

[39] Bower JE. Cancer-related fatigue–mechanisms, risk factors, and treatments. Nat Rev Clin Oncol. (2014) 11:597–609. doi: 10.1038/nrclinonc.2014.127 25113839 PMC4664449

[40] Marsland AL Walsh C Lockwood K John-Henderson NA. The effects of acute psychological stress on circulating and stimulated inflammatory markers: A systematic review and meta-analysis. Brain Behav Immun. (2017) 64:208–19. doi: 10.1016/j.bbi.2017.01.011 PMC5553449 28089638

[41] McDonald TL Hung AY Thomas CR Wood LJ. Localized external beam radiation therapy (EBRT) to the pelvis induces systemic IL-1Beta and TNF-alpha production: role of the TNF-alpha signaling in EBRT-induced fatigue. Radiat Res. (2016) 185:4–12. doi: 10.1667/RR14072.1 26720802 PMC4733565

[42] Fontes F Pereira S Costa AR Gonçalves M Lunet N. The impact of breast cancer treatments on sleep quality 1 year after cancer diagnosis. Support Care Cancer. (2017) 25:3529–36. doi: 10.1007/s00520-017-3777-6 28623402

[43] Vignali DA Collison LW Workman CJ. How regulatory T cells work. Nat Rev Immunol. (2008) 8:523–32. doi: 10.1038/nri2343 PMC2665249 18566595

[44] Dembic Z. On integrity in immunity during ontogeny or how thymic regulatory T cells work. Scand J Immunol. (2019) 90:e12806. doi: 10.1111/sji.12806 31276223

[45] Bower JE Ganz PA Aziz N Fahey JL Cole SW. T-cell homeostasis in breast cancer survivors with persistent fatigue. J Natl Cancer Inst. (2003) 95:1165–8. doi: 10.1093/jnci/djg0019 12902446

[46] Zhang Z Yu Q Zhang X Wang X Su Y He W. Electroacupuncture regulates inflammatory cytokines by activating the vagus nerve to enhance antitumor immunity in mice with breast tumors. Life Sci. (2021) 272:119259. doi: 10.1016/j.lfs.2021.119259 33636172

[47] Liu S Wang ZF Su YS Ray RS Jing XH Wang YQ. Somatotopic organization and intensity dependence in driving distinct NPY-expressing sympathetic pathways by electroacupuncture. Neuron. (2020) 108:436–450.e7. doi: 10.1016/j.neuron.2020.07.015 32791039 PMC7666081

[48] Liu S Wang Z Su Y Qi L Yang W Fu M. Author Correction: A neuroanatomical basis for electroacupuncture to drive the vagal-adrenal axis. Nature. (2022) 601:E9. doi: 10.1038/s41586-021-04290-9 34992295

[49] Jin X Jin L Jin GY. The anti-inflammatory effect of acupuncture and its significance in analgesia. World J Acupuncture-Moxibustion. (2019) 29:1–6. doi: 10.1016/j.wjam.2019.03.003

[50] Lin LL Tu JF Wang LQ Yang JW Shi GX Li JL. Acupuncture of different treatment frequencies in knee osteoarthritis: a pilot randomized controlled trial. Pain. (2020) 161:2532–8. doi: 10.1097/j.pain.0000000000001940 32483056

[51] Zhan J Qin W Zhang Y Jiang J Ma H Li Q. Upregulation of neuronal zinc finger protein A20 expression is required for electroacupuncture to attenuate the cerebral inflammatory injury mediated by the nuclear factor-kB signaling pathway in cerebral ischemia/reperfusion rats. J Neuroinflamm. (2016) 13:258. doi: 10.1186/s12974-016-0731-3 PMC5048665 27716383

[52] Liu W Zhong B Wagner RW Garcia MK McQuade JL Huang W. Systematic review and meta-analysis of acupuncture for modulation of immune and inflammatory markers in cancer patients. Integr Cancer Ther. (2024) 23:15347354241302072. doi: 10.1177/15347354241302072 39663880 PMC11635873

[53] Sun H Zhang B Qian HH Chen ZC. Effect of warm-needle moxibustion intervention on immune function and intestinal flora in patients after colorectal cancer radical operation. Acupuncture Res. (2021) 46:592–7. doi: 10.13702/j.1000-0607.200647 34369680

[54] Wang T Tan JB Yao LQ Huilin C Zhao I Eliseeva S. Effects of somatic acupoint stimulation on anxiety and depression in cancer patients: An updated systematic review of randomized controlled trials. Complement Ther Clin Pract. (2023) 51:101735. doi: 10.1016/j.ctcp.2023.101735 36812735

[55] Han XY Lu Y Jiao H Mei NN Wang J. Effect of Electroacupuncture at Zusanli (ST36) on Pain and Inflammatory Stress Response after Mixed Hemorrhoids Surgery. Shanghai J Acupuncture Moxibustion. (2021) 40:14181423.

[56] Yu QY Sun L Jiang HL Wang Y Zhao BC Yang XJ. Impact of electro-acupuncture on pubescent CRS rats: expressions of TNF-α and IL-1β at different time-points. J Clin Acupuncture Moxibustion. (2017) 33:56–60.

