# Supplementary material for: The effect of Time-Acupoints-Space Acupuncture on fatigue in postoperative chemotherapy patients with breast cancer: a randomized controlled trial
Source: Front Oncol. 2025 Mar 3;15:1518278. doi: 10.3389/fonc.2025.1518278 (PMC11911492; doi:10.3389/fonc.2025.1518278)
Supplement: Supplementary file 2 [file DataSheet2.docx]

**Supplementary Material 2**

Acupoints used in the ATAS group

| **Acupoint** | **Location** |
| --- | --- |
| SI11(Tianzong) | In the region of scapula, in the depression of the center of the subscapular fossa, level with the 4th thoracic vertebra. (needled bilaterally) |
| GV14(*Dazhui*) | On the posterior midline, in the depression below the spinous process of the 7th cervical vertebra. |
| BL18(*Ganshu*) | Level with the lower border of the spinous process of the 9th thoracic vertebra,1.5 *cun* lateral to the posterior midline. (needled bilaterally) |
| GV4(*Mingmen*) | On the posterior midline, in the depression below the spinous process of the 2nd  lumbar vertebra. |
| BL20(*Pishu*) | On the back, level with the lower border of the spinous process of the 11th thoracic vertebra,1.5 *cun* lateral to the posterior midline. (needled bilaterally) |
| GV1(*Changqiang*) | The central point between the tip of the coccyx and the anus below the tip of the coccyx |
| ST8(*Touwei*) | 0.5 *cun* straight above the corner of the anterior hairline, 4.5 *cun* lateral to the anterior midline of the forehead. (needled bilaterally) |
| GV20(*Baihui*) | 7 *cun* directly above the midpoint of the posterior hairline, or at the junction of the midline at the vertex with the line joining the two ear apexes. |
| EX-HN5(*Taiyang*) | In the depression about one finger-breadth posterior to the midpoint between the lateral end of the eyebrow and the outer canthus. (needled bilaterally) |
| GV23(*Shangxing*) | 1 *cun* directly above the midpoint of the anterior hairline. |
| LI4(*Hegu*) | On the dorsum of the hand, between the 1st and 2nd metacarpal bones, approximately in the middle of the 2nd metacarpal bone on the radial side. (needled bilaterally) |
| GV29(*Yingtang*) | Midpoint between the medial ends of the two eyebrows. |
| LR14(*Qimen*) | Directly below the nipple, at the 6th intercostal space, 4 *cun* lateral to the anterior midline.(needled bilaterally) |
| CV17(*Danzhong*) | On the anterior midline, level with the 4th intercostal space, or at the midpoint between the nipples. |
| ST25(*Tianshu*) | 2 *cun* lateral to the umbilicus. (needled bilaterally) |
| CV7(*Yinjiao*) | On the anterior midline, 1 *cun* below the umbilicus. |
| ST36(*Zusanli*) | 3 *cun* below *Dubi*(ST35), one finger width lateral to the anterior crest of the tibia.(needled bilaterally) |
| CV4(*Guanyuan*) | On the anterior midline, 3 *cun* below the umbilicus. |

Acupoints used in the Sham acupuncture group

| **Non-acupoint** | **Location** |
| --- | --- |
| Non-acupoint 1 | GB21(*Jianjing*) outside 1.5 *cun*(needled bilaterally). GB21(*Jianjing*): Midpoint linking the line between GV14(*Dazhui*) and the shoulder acromion. |
| Non-acupoint 2 | BL25(*Dachangshu*) outside 0.5 *cun*(needled bilaterally). BL25(*Dachangshu*): Level with the lower border of the spinous process of the 4th lumbar vertebra,1.5 *cun* lateral to the posterior midline. |
| Non-acupoint 3 | EX-HN5(*Taiyang*) lower 0.5 *cun*(needled bilaterally). EX-HN5(*Taiyang*): In the depression about one finger-breadth posterior to the midpoint between the lateral end of the eyebrow and the outer canthus. |
| Non-acupoint 4 | LU2(*Yunmen*) above 1.5 *cun*(needled bilaterally). LU2(*Yunmen*): On the upper lateral chest, superior to the coracoid process of the scapula, in the depression of the infraclavicular fossa, 6 *cun* lateral to the anterior midline. |
| Non-acupoint 5 | TH13(*Naohui*) outside 1 *cun*(needled bilaterally). TH13(*Naohui*): On the lateral aspect of the arm, on the line connecting the olecranon and TH14(*Jianliao*), on the posterior and inferior border of the deltoideus. |
| Non-acupoint 6 | ST24(*Huaroumen*) outside 1 *cun*(needled bilaterally). ST24(*Huaroumen*): On the upper abdomen,1 *cun* above the center of the umbilicus, 2 *cun* lateral to the anterior midline of the abdomen. |
| Non-acupoint 7 | GB31(*Fengshi*) outside 1.5 *cun*(needled bilaterally). GB31(*Fengshi*): On the midline of the lateral aspect of the thigh, 7 *cun* superior to the popliteal crease. |
| Non-acupoint 8 | ST40(*Fenglong*) outside 1.5 *cun*(needled bilaterally). ST40(*Fenglong*): 8 *cun* above the external malleolus, two finger widths lateral to the anterior crest of the tibia. |
